# Perspectives on health and well-being in nursing

**DOI:** 10.3402/qhw.v9.23026

**Published:** 2014-04-08

**Authors:** Henrika Jormfeldt

**Affiliations:** Halmstad University, Sweden

*As a Guest Editor of the* International Journal of Qualitative Studies on Health and Well-being*'s special edition on perspectives on health and well-being in nursing, it is my wish to present four original articles embracing some essential core aspects of nursing science irrespective of their specialization. They represent different aspects of qualitative research that focus on; the challenge of integrating core concepts of health into mental health nursing praxis, the experiences in psychiatric rehabilitation from the perspective of both patients and their relatives, and the nurses’ experiences of giving support to patients during the transition to hospital-bound hemodialysis. The common basis for the articles is the authors’ ambition in their work of generating nursing knowledge in terms of core elements for the provision of health and well-being among individuals with a need for nursing care*.

The World Health Organization (1991) has defined health potential as involving both physical and mental health seen in the context of an individual's development throughout his or her life. However, outcome measures in health care, as well as in nursing, have instead frequently and unfortunately focused on the absence of symptoms of diseases, in line with a biomedical view of health (Jormfeldt, 2011). An overall aim in nursing science is the development of strategies for supporting patients’ ability to make informed choices regarding their own health and life situation. The goal of nursing care is accordingly to consider and support far more complex processes than just the treatment and cure of illness or disease. Nurses from all specialties have through their caring assignments a responsibility for facilitating their patients’ empowerment. Mental health nurses’ ability to integrate essential concepts into clinical praxis has been described as being dependent on the nurse's ability to assess the patient's needs in order to support patient empowerment and to deal with issues of organizational imbalance of power (Jönsson et al., 2014). A common issue in the development of nursing knowledge across different nursing fields is to consider how to include processes of transition, such as patient empowerment. The process of growth and subjectively experienced health, which has been labeled transition, is most often stimulated through the communication of thoughts and needs (Skärsäter & Willman, 2006). Emotional support has been shown to be of utmost importance in nursing care for the patients during their transition to hospital-bound hemodialysis (Sturesson & Ziegert, 2014). The concept of “transition” is closely related to health and well-being because the meaning of the term involves psychological processes in which the patients adapt to a changing reality. Transition is accordingly a main concern in nursing irrespective of specialization as it involves processes of movement from one state, condition, or situation to another. A crucial aspect regarding the transition toward health and well-being in mental health nursing has been labeled as increased self-understanding, which is achieved through the possibility of verbalizing desires in a trusting relationship (Jormfeldt, Svensson, Hansson, & Svedberg, 2014a). Another essential aspect of nursing irrespective of specialization is the involvement of relatives in the care to assist recognition of the patient's needs. Patients in mental health care are sometimes unable to verbalize their needs and desires, and relatives may have an important role in recognizing and communicating when clients’ needs are insufficiently acknowledged by the nurse (Jormfeldt, Svensson, Hansson, & Svedberg, 2014b). In order to advance nursing care and in turn to enhance health and well-being among patients and their relatives, nursing knowledge has to be gathered, used, and evaluated regarding its capability to support transition toward health and well-being. One of the common main issues in all of the presented articles irrespective of specialization is thus the nurse's ability to provide emotional support to help the patient to verbalize his or her needs and desires.

These articles show that one essential and common part of nursing knowledge irrespective of nursing specialization is the nurses’ ability to provide emotional support. Equality in decision-making processes is achieved through a genuine dialogue aiming to support the patients’ personal resources and insights. The revealed importance of emotional support may be a necessary prerequisite for the patient's development in his or her ability to make decisions in accordance with individual preferences. The presented articles offer core aspects of perspectives on health and well-being in nursing. Hopefully, they will help the reader gain new and broader understandings of nursing knowledge and greater insights into what health and well-being is and how it can be supported through nursing care irrespective of specialization.

*Henrika Jormfeldt* Halmstad University, Sweden
